# The Association between Fatty Liver Index and Lower Limb Arterial Calcification in Patients with Type 2 Diabetes Mellitus

**DOI:** 10.31083/j.rcm2510362

**Published:** 2024-10-10

**Authors:** Peibiao Mai, Qilong Li, Sijin Li, Chunhong Wang, Shuwan Xu, Kun Zhang, Niansang Luo

**Affiliations:** ^1^Department of Cardiology, The Eighth Affiliated Hospital of Sun Yat-sen University, 518000 Shenzhen, Guangdong, China; ^2^Department of Cardiology, Sun Yat-sen Memorial Hospital, 510000 Guangzhou, Guangdong, China; ^3^Department of Cardiology, The Seventh Affiliated Hospital of Sun Yat-Sen University, 518107 Shenzhen, Guangdong, China

**Keywords:** type 2 diabetes mellitus, fatty liver index, lower limb arterial calcification, non-alcoholic fatty liver disease, peripheral arterial disease

## Abstract

**Background:**

Peripheral arterial calcification is a prevalent condition in patients with type 2 diabetes mellitus (T2DM), resulting in lower-limb amputation and reduced life quality. Non-alcoholic fatty liver disease (NAFLD), which can be simply evaluated using the fatty liver index (FLI), is closely associated with T2DM development. In this study, we aimed to explore the relationship between FLI and lower limb arterial calcification (LLAC) in T2DM patients and to reveal the value of T2DM patients with NAFLD in predicting the occurrence of LLAC.

**Methods:**

A total of 77 T2DM patients with LLAC who underwent comprehensive physical and health examinations, serological examinations, as well as lower limb computed tomography imaging at Sun Yat-sen Memorial Hospital of Sun Yat-sen University between January 2018 and January 2019 were enrolled in this study. The FLI was calculated using body mass index, waist circumference, triglycerides, and γ-glutamyl transferase. Additionally, LLAC was evaluated using computed tomography with the Agatston scoring algorithm. The patients were divided into three groups based on their FLI values: Non-liver disease group (FLI <30, n = 29), borderline-liver disease group (30 ≤ FLI < 60, n = 32), and NAFLD group (FLI ≥60, n = 16). Univariate and multivariate binary logistic regression analyses were employed to investigate the association between FLI and LLAC in T2DM patients. Furthermore, differences in LLAC among groups were analyzed using post-hoc multiple comparisons and ordinal logistic regression model analysis.

**Results:**

Univariate and multivariate analyses showed that age and FLI influenced LLAC severity in T2DM patients. Moreover, T2DM patients in the NAFLD group had significantly lower LLAC scores than those in the Non-liver disease group. The correlation analysis showed that FLI was negatively associated with LLAC scores (R = –0.31, *p* = 0.006), while age was positively associated (R = 0.361, *p* = 0.001).

**Conclusions:**

Our study revealed an inverse relationship between FLI and the degree of LLAC. This indicates that, based on evidence in the current research, NAFLD may not be reliable as a predictor of LLAC in T2DM patients.

## 1. Introduction

Type 2 diabetes mellitus (T2DM) is a complex metabolic disorder defined by dysregulated glucose and lipid metabolism [1]. Peripheral artery disease (PAD) is a manifestation of systemic atherosclerosis, particularly prevalent in patients with T2DM. Studies have shown that PAD is an important influencing factor for the increased morbidity and mortality of cardiovascular disease in this patient population [2, 3]. Lower limb arterial calcification (LLAC) is common in patients with PAD, whereby it is associated with the severity of PAD symptoms and independently associated with increased amputation rates and mortality, which greatly affects the quality of life of this population [4, 5]. Therefore, it is imperative to identify and intervene in the associated risk factors of arterial calcification to enhance the prognosis of patients with T2DM [6].

Epidemiological studies have demonstrated a significant overlap in common risk factors between non-alcoholic fatty liver disease (NAFLD) and diabetes mellitus, with NAFLD being identified as a prevalent complication of T2DM [7, 8, 9]. Existing studies have shown that NAFLD is significantly associated with an increased risk of cardiovascular events in patients with T2DM. Specifically, it is an independent risk factor for other cardiovascular and metabolic indicators such as atherosclerosis and insulin resistance [10, 11]. Furthermore, the histological severity of NAFLD is widely recognized as a surrogate marker of subclinical atherosclerosis. Studies have shown a significant association between the severity of NAFLD and arterial stiffness and endothelial dysfunction [12, 13].

However, the existing literature does not definitively establish the association between NAFLD and LLAC in individuals with T2DM. Consequently, there is a gap in the current research addressing the potential correlation between NAFLD and LLAC in this patient population. To bridge this gap and improve clinical diagnosis and treatment, utilizing the fatty liver index (FLI) as a reliable marker for identifying NAFLD in patients with T2DM is critical. By calculating the patient’s FLI, healthcare providers can effectively and efficiently diagnose the presence of NAFLD, streamlining the diagnostic process for better patient care [14, 15]. In this study, we investigated the correlation between FLI and LLAC in T2DM patients to reveal the predictive value of NAFLD for the occurrence and development of LLAC in T2DM.

## 2. Materials and Methods

### 2.1 Study Population

This was a retrospective, observational, single-center study between January 2018 and January 2019. T2DM patients with suspiciously symptomatic lower limb PAD were enrolled in this study. These patients underwent comprehensive physical and health examinations, including age, gender, height, body weight, waist circumference (WC), smoking status, history of hypertension, coronary heart disease (CHD), stroke, diabetes-related foot disease, duration of T2DM, and anti-diabetic drugs at the time of admission. Body mass index (BMI) = body weight (kg)/height^2^ (m^2^). Hypertension was defined as three documented office systolic blood pressure (SBP) readings ≥140 mmHg and diastolic blood pressure (DBP) ≥90 mmHg on different days. CHD was defined as ≥50% diameter stenosis of coronary arteries by coronary angiography or clinical manifestation of cardiac ischemia [16]. The main inclusion criteria of T2DM were based on the diagnostic guidelines of the 1999 World Health Organization [17]. The main exclusion criteria were (1) type 1 diabetes mellitus; (2) a history of alcohol consumption and lower-limb angioplasty bypass or amputation; (3) recent infection inflammatory disorders or hormonal replacement therapies; (4) serious cardiovascular diseases, renal dysfunction or hepatic diseases; (5) malignancy; (6) disability to complete required measurement. This study protocol conformed to the Declaration of Helsinki ethical guidelines.

### 2.2 Biochemical Measurements

Blood samples were collected after a minimum 8 h overnight fast. Serum fasting plasma glucose (FPG), hemoglobin A1c (HbA1c), total cholesterol (TC), triglycerides (TG), high-density lipoprotein cholesterol (HDL-C), low-density lipoprotein cholesterol (LDL-C), albumin, uric acid (UA), phosphorus, calcium, alanine aminotransferase (ALT), aspartate aminotransferase (AST), γ-glutamyl transferase (GGT), superoxide dismutase (SOD), high-sensitivity C-reactive protein (hs-CRP), creatinine, and blood urea nitrogen (BUN), were measured on a standardized and certified TBA-120 auto-analyzer (Toshiba Medical Systems, Tokyo, Japan) in the central laboratory of our unit. Notably, estimated glomerular filtration rate (eGFR) was calculated using the Chronic Kidney Disease Epidemiology Collaboration equation (CKD-EPI) equation [18]. The triglyceride and glucose (TyG) index was calculated using previously established formulas [19]: loge [(TG × FPG)/2]. The FLI was calculated by the following formula [12]: FLI = e0.953 × y/(1 + e0.953 × y) × 100, where y = loge (TG) + 0.139 × BMI + 0.718 × loge (GGT) + 0.053 × WC – 15.745. Here, TG is expressed as mg/dL; FPG is expressed as mg/dL; BMI is expressed as kg/m^2^; GGT is expressed as U/L; WC is measured in cm. FLI values range from 0 to 100, where a FLI <30 rules out steatosis with a sensitivity of 87% and a specificity of 64%, whereas a FLI ≥60 accurately confirms hepatic steatosis with a sensitivity of 61% and a specificity of 86% [14]. According to the above findings, we divided the T2DM patients into three groups: the non-liver disease group (FLI <30), the borderline-liver disease group (30 ≤ FLI < 60), and the NAFLD group (FLI ≥60).

### 2.3 Measurement of Arterial Calcification Using the LLAC Score

Using standard clinical protocols, patients underwent lower limb computed tomography (CT) imaging on a 64-slice CT scanner (Siemens Somatom Definition AS, Munich, Germany). Image analysis was performed on an Apple Macintosh computer (Apple Inc, Cupertino, CA, USA) using the open-source DICOM viewer (v4, OsiriX Imaging Software, Pixeo SARL, Bern, Switzerland). Using the freely available ‘Calcium Scoring’ plug-in, vascular calcification (based on an attenuation threshold of 130 Hounsfield Units in 3 contiguous voxels, after the method of Agatston) was analyzed on consecutive transaxial slices along the length of the arterial segment, as previously described [20, 21].

For this study, the lower limb arterial tree was defined as from the infrarenal aorta to the ankle in both legs, divided into three anatomical segments: The aortoiliac segment (lowermost renal artery to the distal aspect of the iliac artery), the femoropopliteal segment (common femoral artery to the below knee popliteal artery), and the crural segment (the tibiofibular artery trunk and individual crural vessels down to the ankle joint). The total LLAC score for each patient consisted of the sum of the LLAC score of both legs. Individual leg LLAC scores comprised the sum of the aortoiliac, femoropopliteal, and crural segmental LLAC scores [20].

### 2.4 Statistical Analysis

Continuous variables with a normal distribution were reported as the mean ± standard deviation (SD), with skewed data as the median (interquartile range). Categorical variables were presented as numbers (percentages). Baseline variables among patients with different risk groups defined by FLI were compared using analysis of variance (ANOVA) or Kruskal–Wallis test followed by a least significance difference (LSD) comparison or Pearson chi-square test according to the data types. The group differences between different LLAC degrees were compared using Student’s *t*-test, Mann–Whitney U test, and Pearson chi-square when appropriate. Correlation between FLI, age, and LLAC was performed using Spearman’s analysis. Univariate logistic analysis was used to investigate independent risk factors for LLAC. Data are expressed as the odds ratio (OR) and 95% confidence interval (CI). Data were analyzed using SPSS version 20 (SPSS, Inc, Chicago, IL, USA), and two-sided *p*-values < 0.05 were considered statistically significant.

## 3. Results

### 3.1 Baseline Characteristics of Enrolled T2DM Patients

Demographic characteristics, history of the disease, and biochemical and medication data of the enrolled T2DM patients are shown in Table 1. The mean age was 68.7 ± 9.7 years, and 57 (74.0%) T2DM patients were male. The average BMI was 23.3 ± 3.5 kg/m^2^. The mean SBP and DBP were 143.2 ± 24.4 mmHg and 75.7 ± 11.8 mmHg, respectively. The median duration of T2DM was 10.0 (6.0–14.5) years. The mean TyG index and FLI in these patients were 8.89 ± 0.83 and 35.59 (23.15–49.86), respectively. Regarding medication, 43 (55.8%) patients took metformin, and 52 (67.5%) received insulin therapy.

**Table 1.** **Characteristics of enrolled T2DM patients**.

Variable		Patients (n = 77)
Demographic characteristics		
	Age (y)	68.7 ± 9.7
	Male/Female	57/20
	BMI (kg/m^2^)	23.3 ± 3.5
	WC (cm)	90.2 ± 6.4
	SBP (mmHg)	143.2 ± 24.4
	DBP (mmHg)	75.7 ± 11.8
	Hypertension (n, %)	54 (70.1%)
	CHD (n, %)	19 (24.7%)
	Stroke (n, %)	15 (19.5%)
	Diabetes-related foot disease (n, %)	47 (61.0%)
	Smoking (n, %)	37 (48.1%)
	Duration of T2DM (year)	10.0 (6.0–14.5)
	LLAC score	997.0 (146.5–6530.0)
Biochemical characteristics		
	Calcium (mmol/L)	2.23 ± 0.14
	Phosphorus (mmol/L)	1.14 ± 0.17
	Creatinine (µmol/L)	109.81 ± 33.93
	eGFR (mL/min·1.73 m^2^)	61.35 ± 18.17
	BUN (mmol/L)	6.64 ± 2.77
	UA (µmol/L)	388.22 ± 107.19
	FPG (mmol/L)	7.00 ± 3.68
	HbA1c (%)	8.51 ± 2.05
	Albumin (g/L)	36.35 ± 4.86
	HDL-C (mmol/L)	1.01 ± 0.24
	LDL-C (mmol/L)	2.76 ± 0.82
	TG (mmol/L)	1.39 (0.96–2.01)
	TC (mmol/L)	4.50 ± 1.19
	AST (U/L)	18.00 (14.50–22.50)
	ALT (U/L)	17.00 (11.00–23.50)
	GGT (U/L)	32.00 (19.50–52.50)
	hs-CRP (mg/L)	10.49 (2.71–38.57)
	SOD (U/mL)	110.60 ± 23.87
	TyG index	8.89 ± 0.83
	FLI	35.59 (23.15–49.86)
Medications		
	Metformin (n, %)	43 (55.8%)
	Sulfonylureas (n, %)	38 (49.4%)
	Acarbose (n, %)	22 (28.6%)
	Insulin (n, %)	52 (67.5%)

Values are presented as the mean ± standard deviation (SD) or the median (interquartile range (IQR)). ALT, alanine aminotransferase; AST, aspartate aminotransferase; BMI, body mass index; BUN, blood urea nitrogen; CHD, coronary heart disease; DBP, diastolic blood pressure; eGFR, estimated glomerular filtration rate; FLI, fatty liver index; FPG, fasting plasma glucose; GGT, γ-glutamyltransferase; HbA1c, hemoglobin A1c; HDL-C, high-density lipoprotein cholesterol; hs-CRP, high-sensitivity C-reactive protein; LDL-C, low-density lipoprotein cholesterol; LLAC, lower limb arterial calcification; SBP, systolic blood pressure; SOD, superoxide dismutase; T2DM, type 2 diabetes mellitus; TC, total cholesterol; TG, triglycerides; TyG index, triglyceride and glucose index; UA, uric acid; WC, waist circumference.

### 3.2 Characteristics in Different FLI Groups

Based on the FLI values, we divided the T2DM patients into three groups: Non-liver disease group (FLI <30, n = 29), borderline-liver disease group (30 ≤ FLI < 60, n = 32), NAFLD group (FLI ≥60, n = 16). As shown in Table 2, T2DM patients in the NAFLD group tended to be younger than those in borderline-liver disease and non-liver disease groups ((62.5 ± 9.2) vs. (68.9 ± 9.4) vs. (71.9 ± 8.8) years; *p* = 0.006). Compared with T2DM patients in the other groups, T2DM patients in the NAFLD group had significantly higher BMI, DBP, serum lipids (TG and TC), and SOD (all *p* < 0.05). In addition, the prevalence of diabetes-related foot disease was significantly lower in the NAFLD group than in the borderline-liver disease and non-liver disease groups (5 (31.3%) vs. 19 (59.4%) vs. 23 (79.3%); *p* = 0.007). Notably, the insulin resistance marker TyG index was significantly higher in the NAFLD group than in the borderline-liver disease and non-liver disease groups ((9.89 ± 0.70) vs. (8.86 ± 0.57) vs. (8.34 ± 0.61); *p* < 0.001). However, there were no significant differences in other disease history, biochemical data, and medication use among the three groups (all *p* > 0.05).

**Table 2.** **Characteristics of enrolled T2DM patients among different FLI groups**.

Variable		Non-liver disease group (n = 29)	Borderline-liver disease group (n = 32)	NAFLD group (n = 16)	*p*-value
Demographic characteristics					
	Age (year)	71.9 ± 8.8	68.9 ± 9.4	62.5 ± 9.2^*#^	0.006
	Male/Female	19/10	26/6	12/4	0.382
	BMI (kg/m^2^)	21.6 ± 2.9	23.1 ± 3.1	26.6 ± 3.1^*#^	<0.001
	WC (cm)	87.9 ± 6.4	92.5 ± 6.7^*^	89.6 ± 4.2	0.018
	SBP (mmHg)	145.4 ± 21.9	140.4 ± 20.9	145.0 ± 34.5	0.694
	DBP (mmHg)	73.5 ± 10.0	73.2 ± 7.1	84.9 ± 17.3^*#^	0.001
	Smoking (n, %)	13 (44.8%)	16 (50.0%)	8 (50.0%)	0.911
	Hypertension (n, %)	21 (72.4%)	20 (62.5%)	13 (81.3%)	0.391
	CHD (n, %)	10 (34.5%)	6 (18.8%)	3 (18.8%)	0.311
	Stroke (n, %)	4 (13.8%)	6 (18.8%)	5 (31.3%)	0.372
	Diabetes-related foot disease (n, %)	23 (79.3%)	19 (59.4%)	5 (31.3%)	0.007
	Duration of DM (year)	11.0 (9.0–15.5)	10.0 (3.3–15)	10.0 (5–11.8)	0.331
	LLAC score	2024.0 (481.5–7317.5)	989.0 (189.3–8168.0)	270.0 (3.3–1020.3)^*^	0.022
Biochemical characteristics					
	Calcium (mmol/L)	2.21 ± 0.15	2.22 ± 0.14	2.27 ± 0.11	0.368
	Phosphorus (mmol/L)	1.15 ± 0.19	1.13 ± 0.17	1.13 ± 0.15	0.847
	Creatinine (µmol/L)	97.00 (80.50–139.00)	107.00 (87.00–124.25)	104.50 (90.75–151.00)	0.492
	eGFR (mL/min·1.73 m^2^)	59.88 ± 17.65	63.60 ± 17.50	59.59 ± 20.97	0.651
	BUN (mmol/L)	7.06 ± 3.28	6.06 ± 2.27	7.02 ± 2.64	0.313
	UA (µmol/L)	403.41 ± 113.43	385.22 ± 89.11	366.69 ± 107.19	0.540
	FPG (mmol/L)	5.30 (4.20–8.45)	6.15 (4.98–7.57)	7.00 (5.53–8.65)	0.090
	HbA1c (%)	8.06 ± 1.90	8.80 ± 2.09	8.76 ± 2.19	0.317
	Albumin (g/L)	35.73 ± 4.86	35.63 ± 5.23	38.91 ± 3.15	0.058
	HDL-C (mmol/L)	1.06 ± 0.24	1.00 ± 0.25	0.98 ± 0.19	0.486
	LDL-C (mmol/L)	2.64 ± 0.86	2.69 ± 0.73	3.12 ± 0.89	0.141
	TG (mmol/L)	0.97 (0.81–1.17)	1.54 (1.12–1.86)^*^	2.78 (1.92–4.95)^*#^	<0.001
	TC (mmol/L)	4.17 ± 1.00	4.37 ± 0.92	5.36 ± 1.58^*#^	0.003
	AST (U/L)	17.00 (13.50–22.00)	18.50 (14.00–24.25)	20.50 (16.00–23.00)	0.559
	ALT (U/L)	12.00 (9.50–24.00)	16.00 (10.00–21.00)	21.00 (190.0–26.75)^*^	0.038
	GGT (U/L)	26.00 (16.50–32.00)	37.00 (21.25–58.50)^*^	46.50 (33.25–87.25)^*^	0.001
	hs-CRP (mg/L)	16.43 (3.99–91.60)	14.98 (2.32–65.25)	5.85 (2.85–15.97)	0.288
	SOD (U/mL)	102.41 ± 21.32	109.91 ± 24.56	126.81 ± 19.48^*#^	0.003
	TyG index	8.34 ± 0.61	8.86 ± 0.57^*^	9.89 ± 0.70^*#^	<0.001
	FLI	19.01 ± 6.24	40.48 ± 7.26^*^	71.09 ± 20.68^*#^	<0.001
Medications					
	Metformin (n, %)	16 (55.2%)	18 (56.3%)	9 (56.3%)	0.996
	Sulfonylureas (n, %)	17 (53.1%)	12 (41.4%)	9 (56.3%)	0.577
	Acarbose (n, %)	7 (24.1%)	9 (28.1%)	6 (37.5%)	0.351
	Insulin (n, %)	20 (69.0%)	21 (65.6%)	11 (68.8%)	0.955

Values are presented as the mean ± standard deviation (SD) or the median (interquartile range (IQR)). ^*^p < 0.05 vs. non-liver disease group and ^#^*p* < 0.05 vs. borderline-liver disease group. ALT, alanine aminotransferase; AST, aspartate aminotransferase; BMI, body mass index; BUN, blood urea nitrogen; CHD, coronary heart disease; DBP, diastolic blood pressure; DM, diabetes mellitus; eGFR, estimated glomerular filtration rate; FLI, fatty liver index; FPG, fasting plasma glucose; GGT, γ-glutamyltransferase; HbA1c, hemoglobin A1c; HDL-C, high-density lipoprotein cholesterol; hs-CRP, high-sensitivity C-reactive protein; LDL-C, low-density lipoprotein cholesterol; LLAC, lower limb arterial calcification; NAFLD, non-alcoholic fatty liver disease; SBP, systolic blood pressure; SOD, superoxide dismutase; T2DM, type 2 diabetes mellitus; TC, total cholesterol; TG, triglycerides; TyG index, triglyceride and glucose index; UA, uric acid; WC, waist circumference.

### 3.3 Comparison of Characteristics in Different LLAC Groups

To explore the potential risk factors of LLAC. We divided the T2DM patients into two groups based on the median LLAC of all the patients: slight calcification (LLAC <1000) and severe calcification (LLAC ≥1000) groups. As shown in Table 3, T2DM patients in the severe calcification group were significantly older than those in the slight calcification group ((72.0 ± 9.3) vs. (65.4 ± 9.0) years; *p* = 0.002). In addition, the prevalence of CHD was significantly higher in the severe calcification group than in the slight calcification group (14 (35.9%) vs. 5 (13.2%); *p* = 0.015). Interestingly, FLI was significantly lower in the severe calcification than the slight calcification group (33.65 ± 19.38 vs. 44.00 ± 20.91; *p* = 0.027). Moreover, except for HDL-C (0.95 ± 0.21 vs.1.09 ± 0.24 mmol/L; *p* = 0.010), TG (1.73 (1.18–2.13) vs. 1.08 (0.80–1.62) mmol/L; *p* = 0.002), ALT (20.00 (12.50–25.50) vs. 12.00 (10.00–22.00) U/L; *p* = 0.047), there were no significant differences of other serum biochemical markers including TyG index and medication use between the two groups (all *p* > 0.05).

**Table 3.** **Characteristics of enrolled T2DM patients in different LLAC groups**.

Variable		Slight calcification group (n = 38)	Severe calcification group (n = 39)	*p*-value
Population characteristics				
	Age	65.4 ± 9.0	72.0 ± 9.3	0.002
	Male/Female	30/8	27/12	0.275
	BMI (kg/m^2^)	23.7 ± 3.6	22.8 ± 3.4	0.266
	WC (cm)	90.8 ± 6.7	89.5 ± 6.2	0.398
	SBP (mmHg)	139.5 ± 21.2	146.9 ± 26.9	0.185
	DBP (mmHg)	76.0 ± 12.0	75.4 ± 11.7	0.841
History				
	Hypertension (n, %)	25 (65.8%)	29 (74.4%)	0.752
	CHD (n, %)	5 (13.2%)	14 (35.9%)	0.015
	Stroke (n, %)	8 (21.1%)	7 (17.9%)	0.421
	Diabetes-related foot disease (n, %)	20 (52.6%)	27 (69.2%)	0.192
	Smoking (n, %)	19 (50.0%)	18 (46.2%)	0.734
	Duration of DM (year)	10.0 (3.0–12.5)	10.0 (6.0–17.0)	0.121
Biochemical characteristics				
	Calcium (mmol/L)	2.23 ± 0.14	2.22 ± 0.14	0.891
	Phosphorus (mmol/L)	1.14 ± 0.20	1.13 ± 0.15	0.841
	Creatinine (µmol/L)	116.45 ± 37.63	103.33 ± 28.93	0.090
	eGFR (mL/min·1.73 m^2^)	59.44 ± 19.32	63.21 ± 17.03	0.366
	BUN (mmol/L)	6.85 ± 3.18	6.43 ± 2.33	0.508
	UA (µmol/L)	387.18 ± 106.96	389.23 ± 108.80	0.934
	FPG (mmol/L)	5.95 (5.12–7.60)	5. 5 (5.12–7.60)	0.614
	HbA1c (%)	8.80 ± 2.28	8.23 ± 1.77	0.220
	Albumin (g/L)	35.52 ± 4.62	37.16 ± 5.01	0.141
	HDL-C (mmol/L)	0.95 ± 0.21	1.09 ± 0.24	0.010
	LDL-C (mmol/L)	2.70 ± 0.79	2.82 ± 0.86	0.532
	TG (mmol/L)	1.73 (1.18–2.13)	1.08 (0.80–1.62)	0.002
	TC (mmol/L)	4.47 ± 1.15	4.54 ± 1.24	0.804
	AST (U/L)	20.00 (14.00–23.75)	18.00 (15.00–22.00)	0.544
	ALT (U/L)	20.00 (12.50–25.50)	12.00 (10.00–22.00)	0.047
	GGT (U/L)	34.50 (23.25–52.50)	29.00 (17.00–53.00)	0.527
	hs-CRP (mg/L)	23.72 (2.44–51.66)	9.44 (2.89–35.77)	0.333
	SOD (U/mL)	108.29 ± 27.46	112.85 ± 19.86	0.406
	TyG index	9.03 ± 0.85	8.74 ± 0.79	0.122
	FLI	44.00 ± 20.91	33.65 ± 19.38	0.027
Medications				
	Metformin (n, %)	23 (60.5%)	28 (71.8%)	0.341
	Insulin (n, %)	24 (63.1%)	28 (71.8%)	0.472

Values are presented as the mean ± standard deviation (SD) or the median (interquartile range (IQR)). ALT, alanine aminotransferase; AST, aspartate aminotransferase; BMI, body mass index; BUN, blood urea nitrogen; CHD, coronary heart disease; DBP, diastolic blood pressure; DM, diabetes mellitus; eGFR, estimated glomerular filtration rate; FLI, fatty liver index; FPG, fasting plasma glucose; HbA1c, hemoglobin A1c; HDL-C, high-density lipoprotein cholesterol; hs-CRP, high-sensitivity C-reactive protein; LDL-C, low-density lipoprotein cholesterol; LLAC, lower limb arterial calcification; GGT, γ-glutamyltransferase; SBP, systolic blood pressure; SOD, superoxide dismutase; T2DM, type 2 diabetes mellitus; TC, total cholesterol; TG, triglycerides; TyG index, triglyceride and glucose index; UA, uric acid; WC, waist circumference.

### 3.4 Independent Predictors for LLAC

**Fig. 1.**
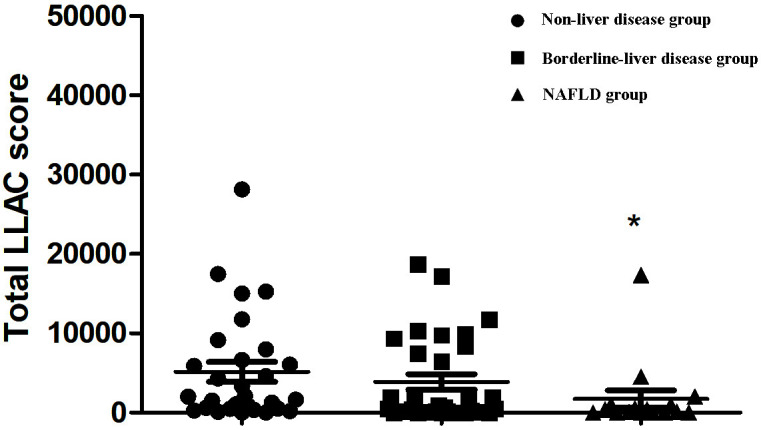
**Comparison of LLAC scores among different groups defined by FLI**. FLI, fatty liver index; LLAC, lower limb arterial calcification; NAFLD, non-alcoholic fatty liver disease. ^*^p < 0.05 vs. non-liver disease group.

**Fig. 2.**
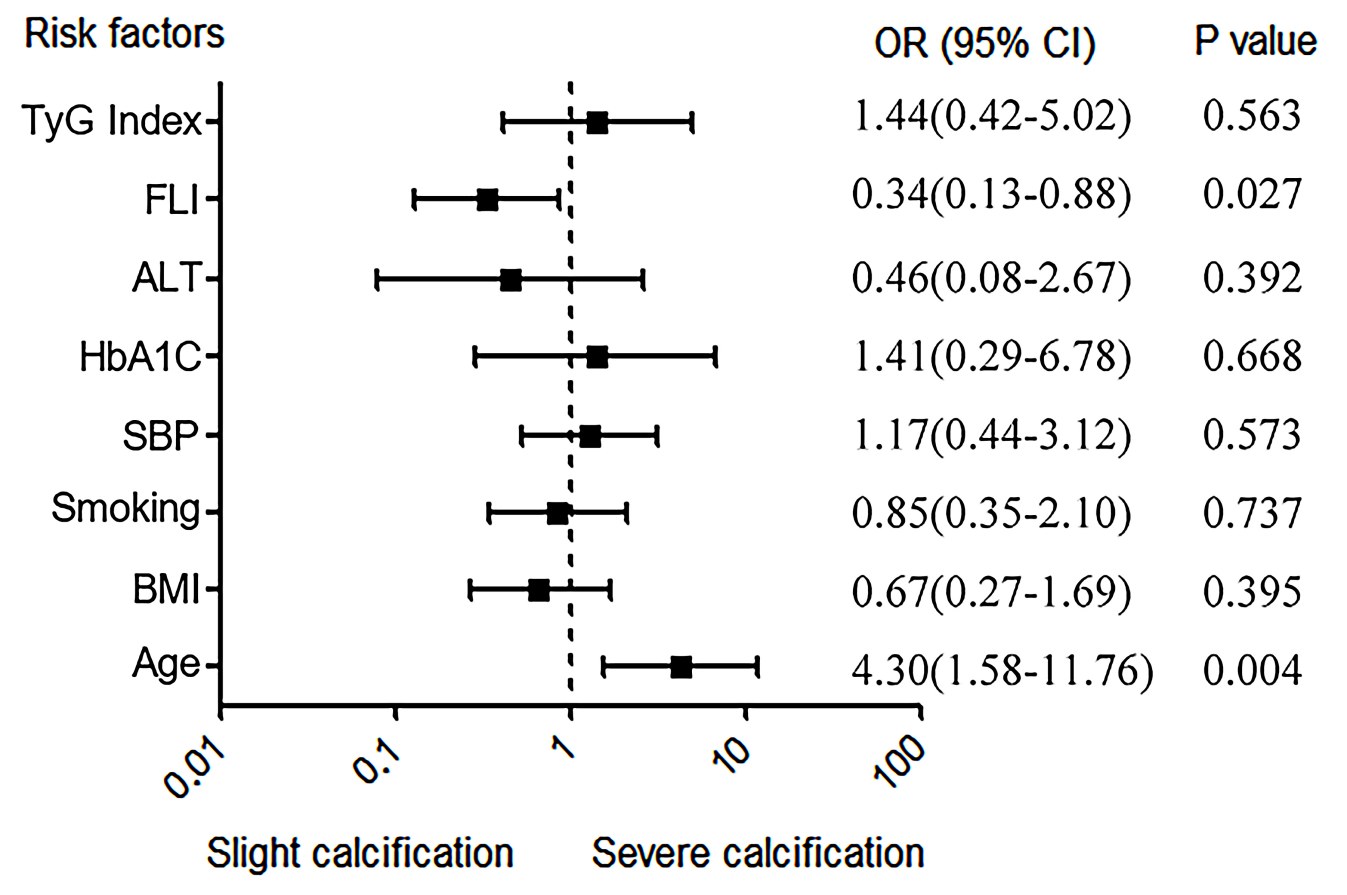
**Independent risk factors for LLAC**. ALT, alanine aminotransferase; BMI, body mass index; HbA1c, hemoglobin A1c; FLI, fatty liver index; LLAC, lower limb arterial calcification; SBP, systolic blood pressure; TyG index, triglyceride and glucose index; OR, odds ratio.

**Fig. 3.**
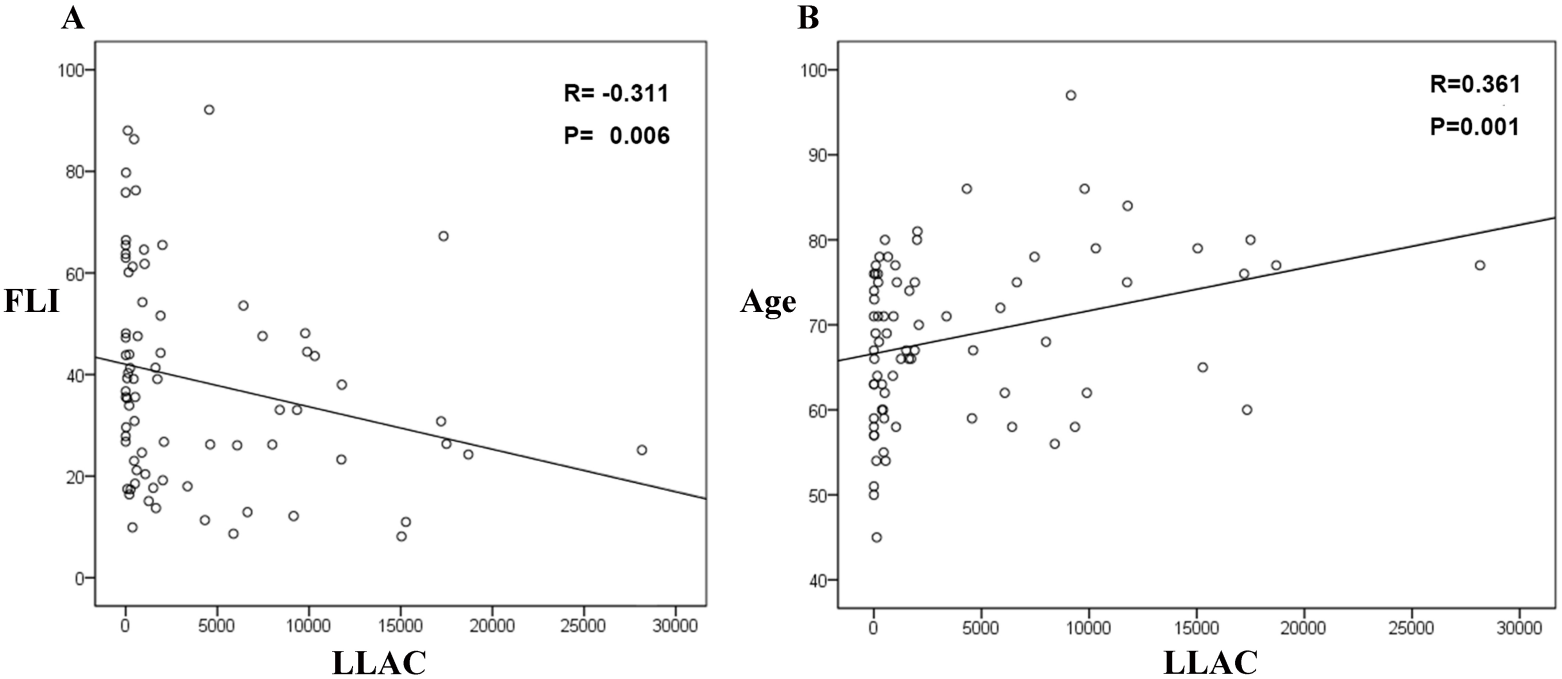
**The associations between LLAC and FLI (A) and age (B)**. FLI, fatty liver index; LLAC, lower limb arterial calcification.

As shown in Fig. 1, the LLAC scores of T2DM patients in the NAFLD group were significantly lower than those in the non-liver disease group (270.0 (3.3, 1020.3) vs. 2024.0 (481.5–7317.5); *p* = 0.018). We further defined the variables (age, BMI, smoking, SBP, HbA1c, ALT, TG, TyG index, and FLI) and grouped the patients into two groups: Age (≥65 or <65 years), BMI (≥25 or <25 kg/m^2^), smoking (yes, no), SBP (≥140 or <140); HbA1c (≥6.5 or <6.5%), ALT (≥40 or <40 U/L), TG (0.31–2.30 or ≥2.30 mmol/L), TyG index (≥8.89 or <8.89), and FLI (≥30 or <30). Then, we analyzed the independent predictors for LLAC, identifying that FLI (OR, 0.339; 95% CI, 0.130–0.883; *p* = 0.027) and age (OR, 4.306; 95% CI, 1.576–11.760; *p* = 0.004) were independent predictors for LLAC (Fig. 2). Then, we analyzed the relationship between FLI, age, and LLAC. FLI was negatively associated with LLAC (r = –0.311, *p* = 0.006; Fig. 3A), while age was positively associated with LLAC (r = 0.361, *p* = 0.006; Fig. 3B).

## 4. Discussion

NAFLD is recognized as a well-established risk factor for cardiovascular events and can be easily and accurately diagnosed by calculating FLI. This study delved into the relationship between FLI and LLAC in T2DM patients, and the findings indicated a negative correlation between FLI levels and LLAC. Moreover, even after controlling for demographic characteristics, previous disease history, blood biochemical indicators, metformin, and insulin history, FLI remained an independent influence factor for the progression of LLAC. Nevertheless, the existing evidence does not yet support that NAFLD predicts the occurrence and progression of LLAC in T2DM patients.

Owing to the shifts in the dietary habits and lifestyles of individuals, NAFLD has emerged as a prevalent global chronic liver condition [22]. Previous studies have confirmed that NAFLD is strongly related to T2DM and metabolic syndrome and is associated with future cardiovascular disease in T2DM patients [7, 8, 9, 23, 24, 25]. A study by Ciardullo *et al*. [26] showed that all-cause mortality and cardiovascular mortality were significantly elevated among patients with PAD, irrespective of whether they were comorbid with T2DM. However, the relationship between NAFLD and PAD remains a topic of debate among experts in the field. Zou *et al*. [27] demonstrated that individuals with both T2DM and NAFLD exhibited a higher prevalence of PAD, characterized by an ankle–brachial index (ABI) <0.9 in either lower limb. This increased occurrence of PAD in NAFLD patients may be attributed to the presence of concurrent metabolic risk factors or an inflammatory response within the body [27]. However, the results of Liu *et al*. [28] did not demonstrate a statistically significant decrease in ABI levels among patients with NAFLD, which is consistent with the findings of our study. In this study, we utilized lower extremity vascular computed tomography angiography (CTA) to diagnose PAD with more precise and objective criteria. In addition, we measured the ABI of patients to assess their vascular health further. However, it was observed that not all patients with LLAC had an ABI less than 0.9. Thus, we propose that the increased prevalence of medial arterial calcification (MAC) in patients with T2DM is the underlying reason for the further reduced extremity blood pressure values and, consequently, elevated ABI value. Therefore, it is essential to note that using an ABI <0.9 as the sole metric for evaluating and diagnosing PAD in these patients may be inaccurate [29, 30]. This discrepancy partly accounts for the difference between our study and the study by Zou *et al*. [27]. In addition, MAC can occur without lipid deposition and inflammatory cell infiltration, which is different from the traditional mechanism of atherosclerotic calcification. Moreover, MAC is more common in lower extremity arteries than in coronary arteries [31, 32], while Salle *et al*. [33]demonstrated the significance of MAC in contributing to lower extremity vascular events in patients diagnosed with T2DM. Although the presence of MAC does not directly cause lumen obstruction, it can decrease the elasticity and compliance of the arterial wall, ultimately leading to atherosclerosis. This condition, in turn, can contribute to the development of CHD or PAD. It is worth mentioning that the factors contributing to the development of MAC and arterial intimal calcification may vary considerably due to the phenotypic switching of vascular smooth muscle cells [31]. Therefore, the presence of calcifications at various anatomical sites may partly explain why NAFLD patients are positively associated with the presence of CHD while being negatively related to the presence of LLAC. In addition, Ponziani *et al*. [34] conducted a recent prospective study and found no significant difference in the incidence of lower extremity atherosclerosis between the NAFLD and control groups. It is worth noting that 16.7% of NAFLD patients in their study suffer from T2DM, which partially confirmed our initial hypothesis [34].

Insulin resistance is a major characteristic of T2DM [35]. Recent research has highlighted the TyG index as a dependable indicator of insulin resistance, with its elevated levels being linked independently to coronary calcification [36]. Our study did not find any association between the TyG index and LLAC. However, we demonstrated that in T2DM patients with high FLI was significantly associated with an increased TyG index. It suggested that NAFLD was significantly associated with insulin resistance in T2DM, consistent with previous data [37, 38]. Moreover, oxidative stress [39] and inflammation [40] are possible mechanisms for LLAC. However, in our study, the levels of hs-CRP were not significantly different among the FLI groups. Regarding the SOD levels, we found high levels of SOD in the high-risk FLI group, although this is contradictory for the patients in this group that have less LLAC. It may attribute to the age factor, whereby the younger individuals in the high FLI group counteract the bad effect of SOD on the vessels. Additionally, we found that T2DM patients with potential NAFLD tended to be younger, which seems to be counterintuitive. However, Forlani *et al*. [41] presented the same conclusion in a cross-sectional study with a much larger sample size. A plausible explanation is that younger people tend to have more unhealthy living habits than older people, such as staying up all night and having a casual approach to diet [42].

This study has certain limitations. Firstly, it is important to note that this research is a single-center cross-sectional study, which may only partially capture the long-term changes and dynamic processes of the population under study. Secondly, since T2DM patients seldom completed the lower limb CT for renal dysfunction or economic reasons (most of them underwent ultrasonography measurement to evaluate lower limb artery), the number of patients we enrolled in this study was relatively small. Thirdly, we used the FLI score to define NAFLD, a diagnostic method lacking liver histological or imagological evidence. Lastly, the LLAC score, which specifically quantifies the calcified portion of atherosclerotic plaques, fails to capture the full extent of the plaque burden due to its exclusion of soft plaques.

## 5. Conclusions

Our study revealed an inverse relationship between FLI and the degree of LLAC. This implies that NAFLD may not be reliable as a predictor of LLAC in T2DM patients. The potential relationship demonstrated in this study warrants further corroboration through multi-center trials and prospective studies with larger sample sizes.

## Availability of Data and Materials

The data underlying this article will be shared upon reasonable request to the corresponding author.
